# Incidence and impact factors of intraoperative loss of light perception under sub-Tenon’s anesthesia in patients with macular diseases

**DOI:** 10.1038/s41433-019-0491-2

**Published:** 2019-06-20

**Authors:** Dezhi Zheng, Zijing Huang, Guihua Zhang, Dingguo Huang, Guoqiao Lin, Weiqi Chen

**Affiliations:** Joint Shantou International Eye Center of Shantou University and The Chinese University of Hong Kong, Shantou, Guangdong, China

**Keywords:** Medical research, Retinal diseases

## Abstract

**Purpose:**

To investigate the incidence and impact factors of intraoperative loss of light perception (LP) under sub-Tenon’s anesthesia in patients with macular diseases.

**Methods:**

Eighty-five consecutive patients received standard phacoemulsification combined pars plana vitrectomy (PPV) under sub-Tenon’s anesthesia. At several checkpoints during the surgery (the end of phacoemulsification, the end of vitrectomy, and the end of surgery), participants were interviewed about whether they had LP or not after removing the influence of contralateral eye and the photo-bleaching effect. In patients treated with retinal photocoagulation, visual experience on laser flashes was evaluated.

**Results:**

Under routine draping, no patients reported loss of LP at all the checkpoints. When the contralateral eye was tightly covered, the rates of LP loss were 84.7%, 97.6%, and 87.1% at the end of phacoemulsification, the end of vitrectomy, and the end of surgery, respectively. When the photo-bleaching effect was also removed, the rates of LP loss were 61.2%, 82.4%, and 56.5% at each checkpoint, respectively, and there were 87.1% (74/85) of patients reporting visual loss in at least one checkpoint. In addition, 76.9% (50/65) of patients could not feel laser flashes during retinal photocoagulation.

**Conclusion:**

Intraoperative loss of LP under sub-Tenon’s anesthesia was a relatively common and reversible event. The conduction block of optic nerve by anesthetic mainly contributed to the visual loss during surgery. Photo-bleaching effect also has some effect on the LP evaluation. Surgeons need to inform and counsel the patients about the intraoperative loss of LP, to prevent any sudden panic attacks in them.

## Introduction

Ocular local anesthesia, including retrobulbar, peribulbar, and sub-Tenon’s anesthesia, has been widely used for intraocular surgeries and proved to be relatively effective and safe. As patients are conscious, they could have various visual experiences during the surgery, such as movement of surgical instruments, changes in light brightness or colors, flashes of light, and loss of light perception (LP) [1, 2]. Evaluation of LP under local anesthesia is considered an important examination for the safety assessment during surgery. Loss of LP during surgery might lead to panic and anxiety for the patients and also bring stress to the surgeon [3, 4].

It was noticed that the incidence rate of LP loss in intraocular surgery under local anesthesia showed large variation in previous studies, presenting with 4.3–25.0% in cataract surgery [3, 5–10] and 6.7–53.8% in pars plana vitrectomy (PPV) [1, 11–13], indicating that some factors might influence the assessment of visual experience. In the present study, we investigated the incidence rate of visual loss and its impact factors in patients with macular hole and epimacular membrane who received phacoemulsification combined PPV surgery under sub-Tenon’s anesthesia.

## Subjects and methods

This study followed the tenets of the Declaration of Helsinki and was approved by a local Research Ethics Committee in Joint Shantou International Eye Center. All participants provided written informed consents. Eighty-five eyes of 85 consecutive patients in Joint Shantou International Eye Center from August 2013 to October 2015 were enrolled. Of the 85 patients, 61 were women, and 50 were diagnosed with macular hole and 35 with epimacular membrane in their operative eye. Patients who showed any one of the followings were excluded from the study: (1) Had a visual acuity of less than Log MAR 1.70 in either eye. (2) Suffered from ocular diseases including glaucoma, optic atrophy, retinal artery occlusion, etc. (3) Had history of cerebral infarction or poorly controlled hypertension. (4) Showed difficulties in cooperating with the investigation during surgery.

**Fig. 1:**
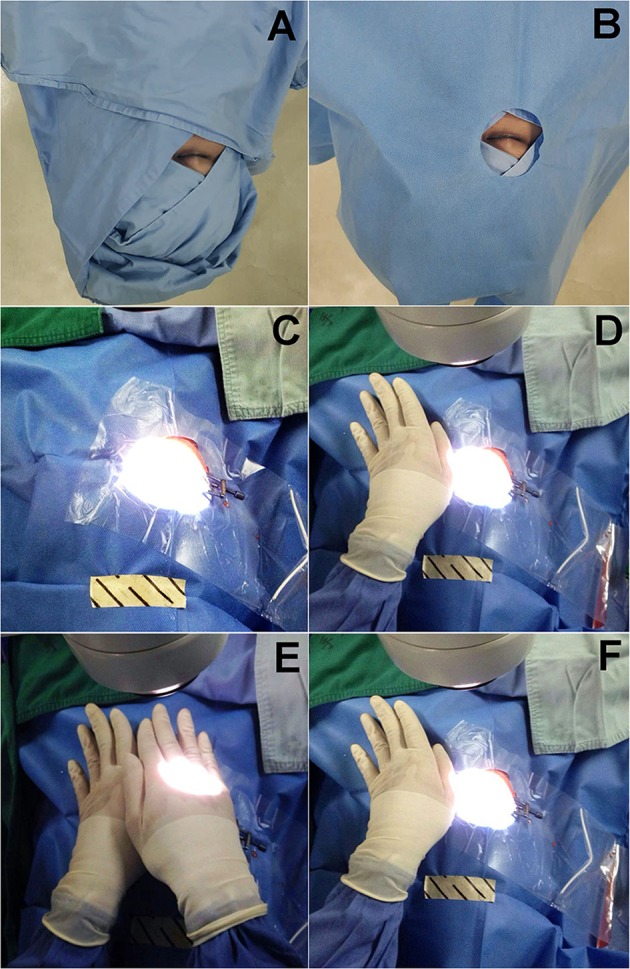
Intraoperative investigation on light perception under different conditions. **a**, **b** Routine eye draping. The contralateral (non-operative) eye was draped with double-layer cloths (**a**) and then a single-layer non-woven fabric (**b**). **c** Investigation of light perception under routine eye draping. **d** Investigation of light perception followed by tightly covering the contralateral eye using five pieces of gauze and the surgeon’s palm on it. **e**, **f** Investigation of light perception after tightly covering the contralateral eye, as well as removing the photo-bleaching effect

All patients received sub-Tenon’s anesthesia and standard phacoemulsification combined PPV surgery by a single surgeon (Dr. Chen). Each patient received oral diazepam at a dose of 2.5 mg for conscious sedation 30 minutes before surgery. After routine skin preparation, the contralateral non-operative eye was draped with double-layer cloths and a single-layer non-woven fabric (Fig. 1a, b). Then sub-Tenon’s anesthesia was performed. In brief, the conjunctiva and Tenon capsule were lifted 4 mm at an inferonasal point from the limbus. An ophthalmic scissor was used to create a small conjunctival and Tenon’s incision. A 2.5-cm curved blunt sub-Tenon’s needle was then inserted into the posterior sub-Tenon’s space along the sclera, and 3.5 ml equivalent mixture of 2.0% lidocaine and 0.75% bupivacaine solution, without epinephrine, was injected into the sub-Tenon’s space. No globe compression was performed. After anesthesia, standard phacoemulsification combined PPV surgery was performed. All surgeries were performed using the same microscope (Opmi Lumero 700, Zeiss, Germany) with the same light intensity (set at 85%). Sixty-five patients also had retinal photocoagulation (532 nm laser, 120–320 mW power, 200 ms duration) after PPV due to retinal degeneration or tears.

At different stages of surgery, patients were interviewed in a real-time manner about whether they had LP or not. The time-points of investigation included the end of phacoemulsification, the end of vitrectomy, and the end of whole surgery. Patients were asked to open both eyes during investigation. At each checkpoint, patients were first inquired if they could feel the light from the microscope with the contralateral eye covered by routine draping (Fig. 1c). Then, they were asked again when their contralateral eyes were covered tightly using five pieces of gauze and the surgeon’s palm to make it completely light-proof (Fig. 1d). After that, patients were investigated again after removing the photo-bleaching effect. Specifically, their operative eyes were covered tightly for 15 s and then uncovered (Fig. 1e, f). During the process of retinal photocoagulation, patients were asked whether they could feel the flashes of laser. In addition, the signs of increased intraocular pressure and ocular ischemia, such as paleness of optic disc and retina and pulsation and narrowing of blood vessels, were investigated during surgery.

The rate of LP loss under different conditions was compared using Pearson Chi-square test or Fisher’s exact test. Logistic regression analysis was used to analyze the correlation between LP loss and other potential factors including age, gender, diagnosis, and operation time. Sample size was calculated using one sample frequency test. The power (1-β) was set as 0.90, and *α* = 0.05. Expected sample size was 30. In this study, 85 patients would be enough to get a statistically significant result. Statistical analysis was performed using SPSS for windows version 20.0 (SPSS Inc., Chicago, IL, USA). Data were shown as mean ± S.D. A *P*-value of less than 0.05 was considered statistically significant.

## Results

Eighty-five patients with macular hole (*n* = 50) and epimacular membrane (*n* = 35) were enrolled. The mean operation duration was 67.5 ± 16.5 minutes (ranging from 44 to 82 minutes). Baseline data were shown in Supplemental Table 1.

**Table 1:** Correlation between the rate of loss of light perception and baseline factors

Conditions	Contralateral eye covered tightly		Contralateral eye covered tightly and photo-bleaching effect removed	
	OR	*P*-value	OR	*P*-value
Gender	0.74	0.67	0.3	0.63
Age	1	0.96	0.97	0.38
Pre-op BCVA	1.27	0.8	2.39	0.33
Diagnosis	0.74	0.67	0.74	0.55
Operation time	0.96	0.41	0.99	0.34

*OR* odds ratio

**Fig. 2:**
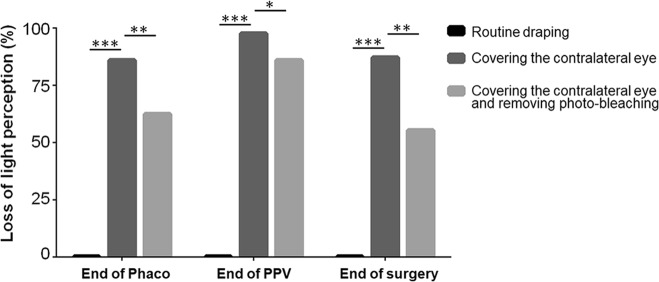
Assessment of intraoperative light perception loss in different stages of surgery under different conditions. Patients received sub-Tenon’s anesthesia and phacoemulsification combined PPV. With contralateral eye draped routinely, all patients had LP. With contralateral eye covered tightly, the rate of visual loss was 84.7, 97.6, and 87.1% at three checkpoints postoperatively. Removal of photo-bleaching effect by covering the operative eye for 15 seconds reduced the rate of intraoperative visual loss to 61.2, 82.4, and 56.5%. Phaco: phacoemulsification. PPV: pars plana vitrectomy. ***P < 0.001, **P < 0.01, *P < 0.05

**Fig. 3:**
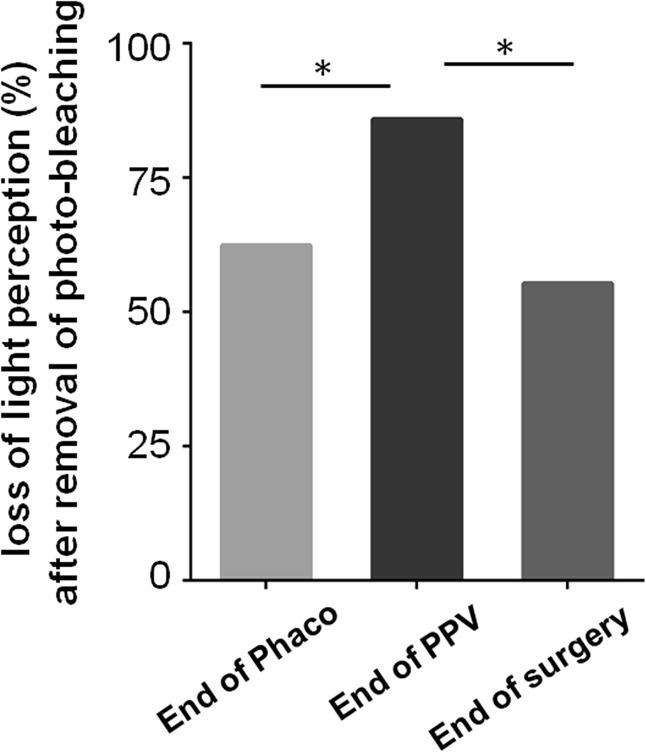
Comparison of rate of visual perception loss during the surgery. The rate of LP loss showed an increase and then declined at the checkpoints after tightly covering the contralateral eye and removing the photo-bleaching effect. Phaco: phacoemulsification. PPV: pars plana vitrectomy. *P < 0.05

Under routine draping (Fig. 1c), no patients reported loss of LP at all the checkpoints. When patients’ contralateral eye was tightly covered (Fig. 1d), the rate of intraocular LP loss was 84.7%, 97.6%, and 87.1% at the three checkpoints (end of phacoemulsification, end of vitrectomy, end of surgery) respectively (Fig. 2). When the photo-bleaching effect was removed by covering the operative eye for 15 s and then uncovered it again (Fig. 1e, f), the incidence rate of LP loss was 61.2, 82.4, and 56.5% respectively, indicating that photo-bleaching effect might affect the LP evaluation (Fig. 2). Because of the physiological visual adaption that long-lasting strong light exposure could reduce visual sensitivity, the visual loss rate after the removal of photo-bleaching effect should be considered as the true loss of LP. When photo-bleaching effect was eliminated, there were still 87.1% (74/85) of participants suffered from visual loss in at least one of three checkpoints, and 43.5% (37/85) even experienced no LP at all three checkpoints. We also noticed that the rate of LP loss showed an initial increase and then decline, with a highest incidence at the checkpoint of PPV procedure (Fig. 3). Logistic analysis showed no correlation in intraoperative LP loss with gender, age, best corrected visual acuity (BCVA), diseases, and operation time (Table 1).

**Fig. 4:**
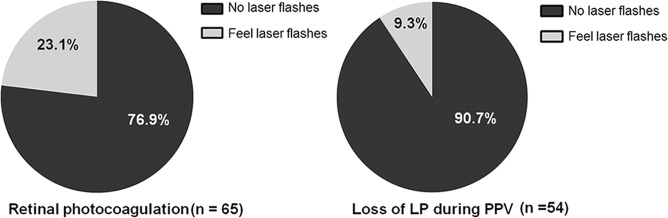
Visual perception of laser flashes during surgery. In patients receiving retinal photocoagulation, 23.1% (15/65) could feel the flashes of laser, while 76.9% (50/65) reported no perception of laser. In patients who received laser therapy and had no light perception at the PPV checkpoint, 9.3% (5/54) could feel the laser flashes during photocoagulation

In addition, of the 65 patients who received retinal photocoagulation after vitrectomy, 76.9% (50/65) failed to feel the flashes of laser during photocoagulation. It was noticed that of the 54 patients who received laser therapy and reported loss of LP at the PPV checkpoint, 9.3% (5/54) were able to feel the laser (Fig. 4), while those who had LP at the end of PPV checkpoint could also feel the laser flashes.

The signs of ocular ischemia, such as paleness of optic disc and pulsation and narrowing of blood vessels, were not found in all the operated eyes, indicating a relatively stable IOP during surgery. During investigation, all participants did not have any discomfort apart from loss of LP during and after the operation. All operations were performed smoothly and no additional intervention was adopted for the visual loss. The visual acuity of the operative eye in all patients restored to LP or better at first postoperative day without any ocular complications.

## Discussion

Ocular local anesthesia has proved to be safe and cost-effective in intraocular surgeries. With increasing popularity of local anesthesia, much attention has been paid to the various intraocular visual experiences of the patients. However, as is reported, whether patients could have light perception from the operative eyes during surgery remain unknown and controversial [14]. The loss of LP in ocular surgery has been once reported, however, with large varied incidence rates. Murdoch [15] reported 3.6% of patients who lost LP during cataract surgery under peribulbar anesthesia, while Tan [1] found 53.8% of patients receiving retrobulbar anesthesia suffered from intraoperative visual loss.

In clinical practice, patients with monophthalmia or absolute glaucoma in their non-operative eye seemed more likely to complaint no LP during ocular surgery [16–18], indicating that the contralateral eye might impact intraoperative visual experience. In this study, we covered the contralateral eye to make it completely light-proof and found a significant number of patients reported loss of LP during surgery. The non-operative eye is generally draped with two or more layers of sterile cloth in ophthalmic surgery. However, due to the light-transparency of cloth and strong surgical illumination, patients could very likely get LP from their non-operative eye. This speculation has been confirmed in another of our study (data not shown), which partially explained why the incidence rate of LP loss varied largely in previous studies, since most investigators might pay less attention to the situation of contralateral eye.

Another factor impacting the light experience was the “photo-bleaching effect”. This effect comes from a physiological visual adaption in which long-lasting strong light exposure could reduce visual sensitivity. It is known that light-induced cone phototransduction depends on the opsin function. Continuous higher levels of illumination will bleach away photopigments, making the outer segment of cones insensitive to light. To remove this effect, we kept the operative eye completely light-proof for 15 s and uncovered it again. As expected, the incidence rate of LP loss showed a significant decline, indicating that the photo-bleaching effect played some role in intraoperative LP loss. We also noticed that after removal of photo-bleaching, the rate of LP loss fell much more in the phaco and end checkpoints than the PPV checkpoint, suggesting that the photo-bleaching might have a greater impact on LP assessment when anesthetic effect was relatively weak. Conversely, when anesthetic effect reached its maximum, the photo-bleaching played a minor role. Since photo-bleaching is a normal physiological phenomenon, the “true” intraocular visual loss should be defined after removing of this effect.

The timing of investigation also influenced the accuracy of results. Most previous studies were retrospective questionnaire-based investigation in which participants were interviewed several hours or one day after surgery [19, 20]. Under local anesthesia, patients might have various visual experiences at different stages of surgery. In addition, since the PPV procedure costs a bit of time, patients could likely get different visual impressions during this period. Therefore, it would be rather difficult for them to accurately recall the visual experience, including whether they had visual loss at some time. In this study, patients received investigation in a real-time manner, and all of them were confirmed conscious before the investigation, which ensured a more reliable result about their visual impression.

Subjective visual experience on intraocular laser therapy has rarely been reported. Because the laser is much brighter than the microscope light, patients are more likely to feel the laser flashes if they have visual perception during surgery. In this study, however, 76.9% (50/65) of patients failed to have laser sensation, further confirming the intraocular loss of LP. In addition, we also noticed that 9.3% (5/54) who lost LP in PPV procedure were able to feel the laser, and three of them finally restored LP at the end checkpoint, indicating the effect of anesthetic was reducing so that patients were able to have visual perception under strong light exposure.

The mechanism of LP loss under local anesthesia has been once discussed. Some researchers believed it was due to conduction block of the optic nerve by the anesthetic [8, 21, 22]. Kumar observed the accumulation of anesthetic solution around the optic nerve after sub-Tenon’s injection [23]. Ramsay found relative afferent pupil block (RAPD), indicating the block of visual pathway, occurred several minutes after local anesthesia and lasted for about 30 min [21]. In this study, consistently, the incidence rate of visual loss increased and then decreased, with a highest rate at the PPV checkpoint, raising a possibility that the nerve block effect of anesthetic was increasing and reached its maximum when PPV was performed. In addition, some patients who suffered from visual loss in PPV procedure could feel the laser flashes during photocoagulation after PPV, probably because the effect of anesthetic was reducing, and that laser was much brighter than microscope light. Furthermore, decrease of amplitude and prolonged latent phase in visual evoked potential (VEP) examination after retrobulbar anesthesia was once reported [24, 25]. These findings provided evidence for the transient visual transduction block as a reasonable explanation for anesthesia-induced visual loss. Some others argued that injection of anesthetic fluid would increase intraocular pressure (IOP), resulting in ischemia of the retina and optic nerve and leading to visual loss [26, 27]. In this study, however, we did not observe paleness of optic disc and retina or pulsation and narrowing of blood vessels which indicated ocular ischemia. In addition, visual function was difficult to restore if retinal ischemia lasted for more than 60 min, while all patients in this study restored to at least LP and their final visual acuity was significantly improved than pre-operation, implicating that retinal ischemia might not be a possible mechanism for the anesthetic-induced visual loss. Other factors include direct injury to the optic nerve by the injection needle [28], which could rarely happen during sub-Tenon’s anesthesia when a blunt needle was used.

Some previous study considered intraoperative loss of LP as anesthetic or surgical complications [29]. In some cases, intraoperative visual loss would lead to suspending or even cancelation of the surgery, and patients were given with supportive care such as oxygen and vasodilatation therapy [30]. In this study, excluding the impact of contralateral eye and the photo-bleaching effect, there were still 87.1% (74/85) of patients suffered from loss of LP at least in one period of the surgery under local anesthesia, which was markedly higher than previous reported. However, importantly, all of them restored to a visual acuity of LP or better in the first postoperative day. Follow-up was also conducted to discharge, and no impaired vision or other ocular complication was observed. The intraoperative loss of LP seemed relatively common and safe than thought without the need of specific intervention.

There were some limitations in our study. First, we covered the operative eye shortly (15 seconds) to remove the photo-bleaching effect because it seemed unreasonable to stop the surgery for quite a long time. The short covering might be unable to completely remove the photo-bleaching effect, raising a possibility that this effect would play a larger role in intraoperative visual loss than expected. Nevertheless, to our knowledge, no previous studies have focused on the impact of photo-bleaching effect and take measures to avoid it. Second, although IOP was maintained at ~30 mmHg during surgery, we did not directly measure the IOP during surgery. In addition, the rate of intraoperative LP loss may differ with local anesthesia techniques. Since anesthetic from retrobulbar or peribulbar injection cannot always reach to muscle pyramid near the optic nerve, these techniques may have longer onset time and lower rate of intraoperative LP loss. Third, the fluctuations in vital parameters during surgery, particularly blood pressure and heart rate, could affect blood perfusion of the brain and eye [31], and therefore might influence patients’ subjective visual perception. Unfortunately, we did not notice the fluctuations in vital parameters at the time when patient reported no light perception, although all the vital parameters were in normal ranges under electrocardiogram monitoring during the whole surgery, with systolic blood pressure of 100 to 160 mmHg, and heart rate of 60–100 beats/min. Further investigation is needed to answer these questions.

In this study, we found sub-Tenon’s anesthesia-induced loss of LP as a relatively common but reversible event. Temporary conduction block of optic nerve by the anesthetic is believed as a reasonable explanation for this phenomenon. The photo-bleaching effect also has some effect on the LP evaluation. Surgeons need to inform and counsel the patients about this event, to prevent any panic attacks from occurring due to undue stress and anxiety.

## Summary

### What was known before

- Tha rate of loss of light perception under local ocular anesthesia was 4.3–25.0% in cataract surgery and 6.7–53.8% in PPV, with large variation in previous studies.
- Whether loss of light perception lead to poor outcomes of the surgery remains unclear.

### What this study adds

- Intraoperative loss of light perception under sub-Tenon’s anesthesia was a relatively common but reversible event.
- The conduction block of optic nerve by anesthetic mainly contributed to the visual loss during surgery.

## Supplementary information

Supp Table 1: Patient demographics
